# Predicting acetabular version in native hip joints through plain x-ray radiographs: a comparative analysis of convolutional neural network model and the current gold standard, with insights and implications for hip arthroplasty

**DOI:** 10.3389/fsurg.2024.1329085

**Published:** 2024-10-15

**Authors:** Ata Jodeiri, Hadi Seyedarabi, Parmida Shahbazi, Fatemeh Shahbazi, Seyed Mohammad Mahdi Hashemi, Seyed Mohammad Javad Mortazavi, Seyyed Hossein Shafiei

**Affiliations:** ^1^ Faculty of Electrical and Computer Engineering, University of Tabriz, Tabriz, Iran; ^2^ Faculty of Advanced Medical Sciences, Tabriz University of Medical Sciences, Tabriz, Iran; ^3^ Orthopaedic Subspecialty Research Center (OSRC), Sina University Hospital, Tehran University of Medical Sciences, Tehran, Iran; ^4^ School of Electrical & Computer Engineering, University of Tehran, Tehran, Iran; ^5^ Joint Reconstruction Research Center, Tehran University of Medical Sciences, Tehran, Iran

**Keywords:** hip joint, acetabulum, acetabular version, artificial intelligence, machine learning, deep learning, convolutional neural network

## Abstract

**Introduction:**

This study presents the development and validation of a Deep Learning Convolutional Neural Network (CNN) model for estimating acetabular version (AV) from native hip plain radiographs.

**Methods:**

Utilizing a dataset comprising 300 participants with unrelated pelvic complaints, the CNN model was trained and evaluated against CT-Scans, considered the gold standard, using a 5-fold cross-validation.

**Results:**

Notably, the CNN model exhibited a robust performance, demonstrating a strong Pearson correlation with CT-Scans (right hip: *r* = 0.70, *p* < 0.001; left hip: *r* = 0.71, *p* < 0.001) and achieving a mean absolute error of 2.95°. Remarkably, over 83% of predictions yielded errors ≤5°, highlighting the model's high precision in AV estimation.

**Discussion:**

The model holds promise in preoperative planning for hip arthroplasty, potentially reducing complications like recurrent dislocation and component wear. Future directions include further refinement of the CNN model, with ongoing investigations aimed at enhancing preoperative planning potential and ensuring comprehensive assessment across diverse patient populations, particularly in diseased cases. Additionally, future research could explore the model's potential value in scenarios necessitating minimized ionizing radiation exposure, such as post-operative evaluations.

## 1 Introduction

Artificial Intelligence (AI) refers to the development of computer systems that can perform tasks that typically require human intelligence. These machines are capable of learning from repetitive experience, recognizing patterns, and making decisions. AI aims to replicate human cognitive functions by using algorithms and computational models. It encompasses a wide range of technologies and applications, from virtual assistants to complex systems that analyze data for medical diagnoses (1, 2). Essentially, AI enables machines to think, learn, and solve problems in ways that were once thought to be exclusive to human intelligence. Utilizing these tools can minimize costs, workload, and reduce inevitable human errors (3).

The adoption of AI in orthopedic practice is increasing, spanning clinical, preoperative, intraoperative, and postoperative phases. In orthopedic surgery, where clinical decisions and image interpretation can be subjective and reliant on reviewer experience, AI offers the potential to mitigate errors, particularly among less experienced practitioners (4). Recent evidence underscores the suitability of hip x-rays for deep learning-based image recognition, showcasing orthopedics’ compatibility with AI advancements (5).

Total hip arthroplasty (THA) has earned the title of “Operation of the Century” and the number of people undergoing THA is increasing. AI has been tried to identify hip implants prior to revision surgery, thus saving significant time, and reducing perioperative morbidity and healthcare cost (6, 7).

The acetabular version (AV) is one of the anatomical features of the hip joint, defined as the angle between the line connecting the most posterior and the most anterior edge of the acetabulum in the axial plane and the sagittal plane of the body. Acetabular anteversion with an average angle between ∼15–20 degrees in a native hip plays a crucial role in the biodynamics and stability of the hip joint (8, 9). AV abnormalities can lead to several problems, such as osteoarthritis, dysplasia, impingement, dislocation, and even posterior wall fractures. Research suggests that measuring the version of the acetabulum is essential in patients who experience hip pain, especially in juveniles (10).

In the context of hip arthroplasty preoperative planning, determining AV is important in order to achieve optimal restoration. The issue has grown in importance since studies show abnormal version restoration is related to recurrent dislocation followed by revision surgery and increased component wear (11–13).

While the gold standard method for assessing AV is CT-Scan, concerns about radiation exposure and cost have led to attempts to replace it with plain x-ray radiographs (14, 15). However, the debate continues due to the perceived disadvantages of plain x-rays, such as being time-consuming, operator-dependent, and inaccurate (16).

The purpose of this paper is to describe and evaluate a fully automated Convolutional Neural Network (CNN) model (17), that has been developed and trained to accurately estimate the AV in the native hip without any operator dependency.

## 2 Methods and materials

### 2.1 Case selection

After obtaining ethical approval and the hospital’s research committee clearance (IR.TUMS.SINAHOSPITAL.REC.1399.034), we utilized our local medical institution’s prospective data registry of patients from 2018–2020 who were admitted due to trauma and, as per institutional guidelines, underwent pelvis CT-Scan as well as pelvic Anteroposterior (AP) radiography.

The inclusion criteria for our study were as follows:

1. Otherwise, healthy, multiple trauma patients
2. Availability of both AP pelvis radiograph and CT-scan

Below are the exclusion criteria that were applied:

1. Any evidence of fracture or dislocation of the acetabulum, femoral head or neck, and the pelvic ring in either CT-Scan or x-ray
2. Previous injuries or interventions to the hip area
3. Abnormal or nonstandard x-ray radiographs or CT-Scan
4. History or evidence of diseases related to the hip joint e.g., osteoarthritis, rheumatologic diseases, and so forth

Among 1,800 cases, 300 consecutive patients who matched our inclusion and exclusion criteria were included for training and testing the CNN model.

### 2.2 Data collection

All 300 cases x-ray images and CT-Scans were downloaded from the hospital’s picture archiving and communication system (PACS). These images were reviewed by a trained last-year medical student (S.M.M.H.) under the supervision of a fellowship-trained hip surgeon (S.H.S.). Additional variables such as age, gender, and reason for hospitalization were extracted from the hospital information system (HIS).

**Figure 1:**
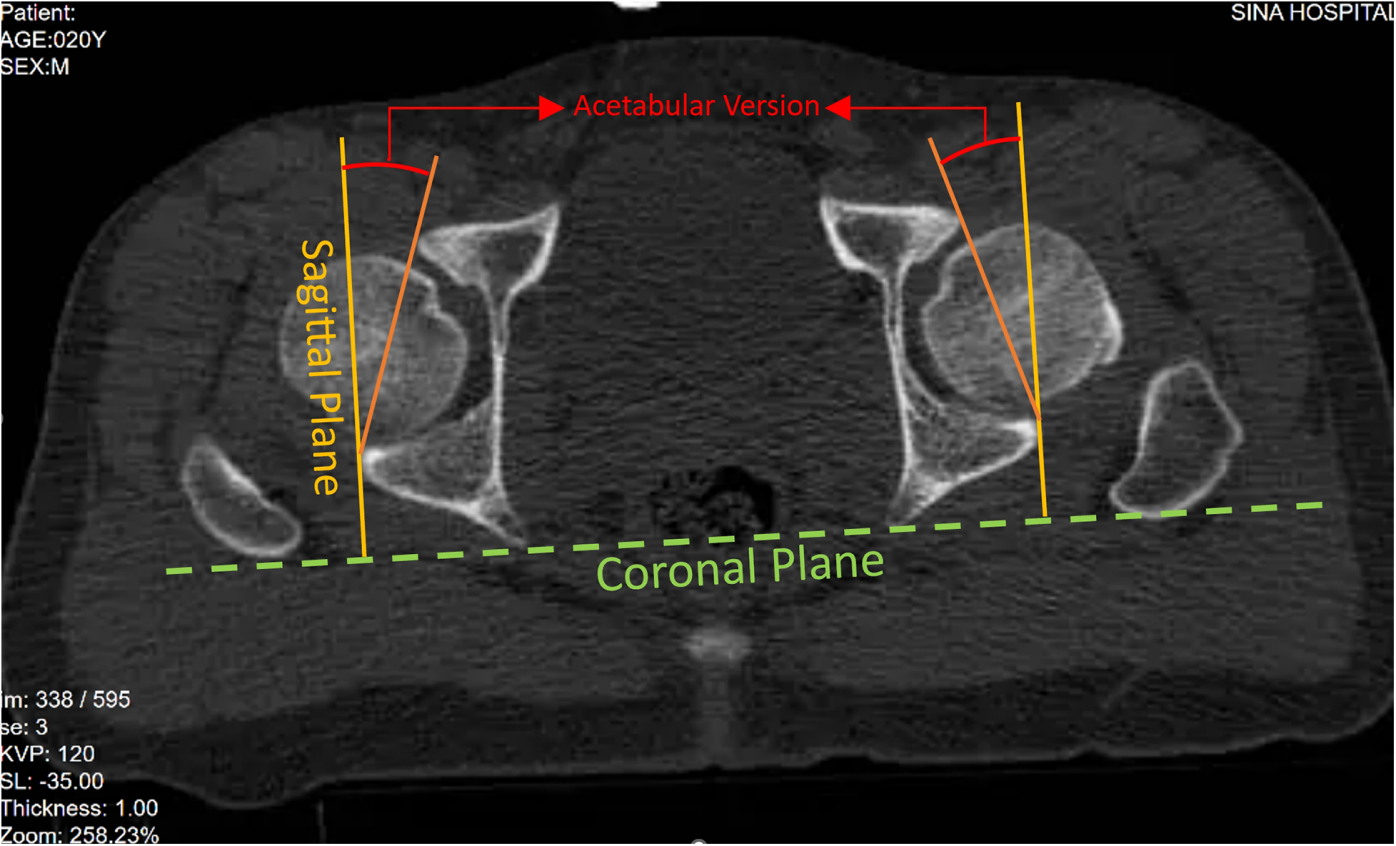
Measurement of acetabular version in equatorial plane of acetabulum CT-Scan.

A Philips Ingenuity Flex 16-slice CT-Scan (Philips Medical Systems Ltda, Lagoa Santa, MG-Brazil) with fine 2 mm slices was used, with the patient positioned supine. The AV was manually measured in the horizontal slice precisely at the middle of the acetabulum (equatorial plane) for each side using MicroDicom v0.7.7. This measurement was taken as the angle between the line connecting the most posterior and anterior edges of the acetabulum in the axial plane and the sagittal plane of the body (18) (Figure 1).

AP plain x-rays were taken in the supine position using Varian Digital equipment (DRGEM Corporation, Gwangmyeong-si, South Korea), with the beam centered on the pubic symphysis. Following the methodology outlined by Lim SJ. et al. for the evaluation of standard plain hip radiographs, the symmetry of the obturator foramen and iliac wings, the alignment of the sacrum and pubic symphysis were analyzed, and the appropriate positioning of the beam was validated by measuring the distance between coccyx tip and the pubic symphysis (19). Through these analyses, we established the baseline normalcy for the AP radiographs, while eliminating radiographs with tilt or rotation.

All records were inputted into an Excel sheet for analysis.

### 2.3 Convolutional neural network model training

#### 2.3.1 Neural network (NN) architecture

CNNs represent a sophisticated framework composed of two fundamental components essential for image processing tasks: a NN architecture inspired by the intricate organization of the human brain, and convolution, a pivotal algorithmic technique employed for feature extraction. The NN aspect serves as the foundation for data processing, meticulously analyzing input data to extract meaningful patterns and representations. In tandem, convolution, through its application of mathematical operations on the input data, plays a crucial role in identifying and highlighting relevant features within the input images. By convolving learnable filters across the input data, CNNs efficiently capture spatial hierarchies of features, facilitating robust pattern recognition capabilities. This process significantly reduces the computational burden associated with traditional methods, enabling CNNs to effectively process large-scale image datasets with improved accuracy and efficiency. Thus, the integration of NN principles with convolutional techniques underpins the remarkable performance of CNNs in various medical imaging tasks, ranging from disease diagnosis to medical image analysis and beyond (15, 20).

#### 2.3.2 End-to-end learning approach

Our training methodology embraces an “end-to-end” strategy, where in raw data encompassing unprocessed AP pelvic x-ray DICOM radiographs, demographic information such as age and gender, and the AV angle measured via CT-Scan, which were seamlessly inputted into the NN. This approach eliminates the need for manual intervention, as the NN autonomously refines its parameters through iterative adjustments, optimizing its performance based solely on the provided dataset.

#### 2.3.3 Data preprocessing

Initially, a dataset of 300 standard AP pelvic x-ray images, each labeled with the corresponding acetabular angle measured by CT-Scan, age, and gender, was assembled. These radiographs were anonymized and standardized in terms of size (1,024 × 1,024), contrast, brightness, and removal of irrelevant elements. To augment the dataset and improve training, techniques such as vertical mirroring and data augmentation (including vertical and horizontal shifting and zooming) were employed.

#### 2.3.4 Model architecture and training

To establish a robust foundation, a pre-trained VGG16 CNN model, developed by the Visual Geometry Group at the University of Oxford was employed, initially trained on diverse non-medical image datasets, as a starting point. Leveraging transfer learning, this model was fine-tuned using the supplied radiographic data, adapting its parameters to the intricacies of medical imaging. Furthermore, to prioritize pertinent regions while disregarding artifacts or abnormalities, an attention mechanism was integrated into the model architecture. The training regimen spanned 1,000 epochs, with periodic adjustments to the training algorithm strategically implemented to iteratively enhance model performance and optimize its capacity for medical image analysis.

#### 2.3.5 Performance evaluation

**Figure 2:**
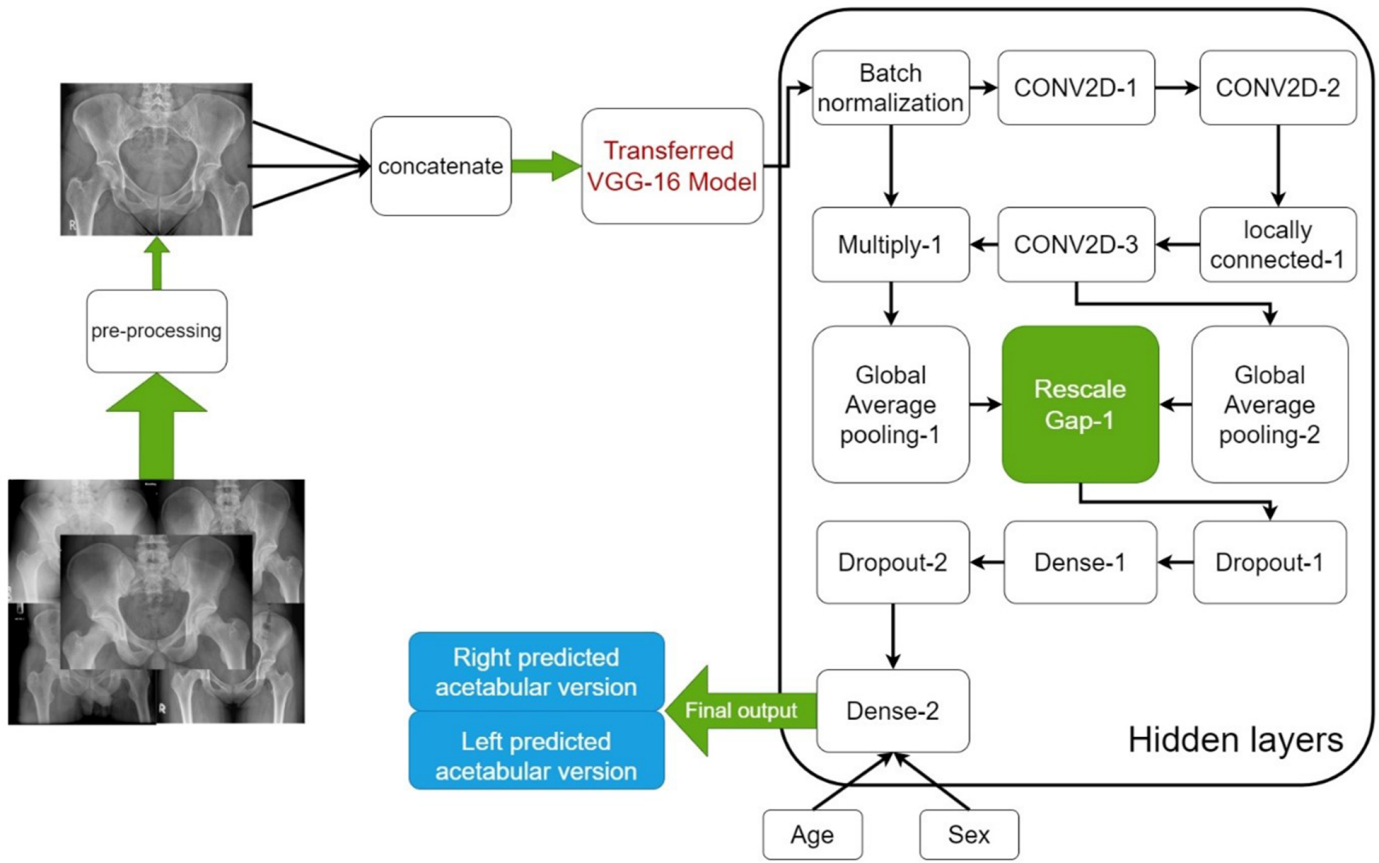
Block diagram of the proposed model. CONV2D-1: 2-Dimensional Convolutional Layer with 1 filter, CONV2D-2: 2-Dimensional Convolutional Layer with 2 filter, CONV2D-3: 2-Dimensional Convolutional Layer with 3 filter.

Throughout training, performance metrics such as Mean Squared Error and the Adaptive Movement Estimation (ADAM) algorithm were employed to assess model accuracy and guide adjustments. Additionally, a 5-fold cross-validation technique was utilized to mitigate dataset limitations and prevent bias. This involved dividing the dataset into five randomly selected groups, with 80% of images used for training and 20% for evaluation. Notably, none of the training subjects were used for testing to maintain model integrity. A schematic view of proposed method can be seen in (Figure 2).

### 2.4 Data analysis

The data were imported and analyzed using IBM SPSS software version 25. Categorical variables were summarized by their frequencies, while continuous variables were summarized using either the mean and standard deviation (SD) or the median and range, depending on the distribution of the data.

For comparing the mean AV between the CNN model and CT-Scan, a paired sample *t*-test was utilized, contingent upon the fulfillment of assumptions such as normality and homogeneity of variances. Additionally, an independent sample *t*-test was employed to compare AV across different genders. Results were expressed as the standard mean difference (SMD) with a 95% confidence interval (CI).

Skewness and Kurtosis tests were performed to evaluate the normality of the results. The correlation between predicted and actual AV values was evaluated using Pearson correlation analysis. The Mean absolute Error (MAE), representing the average absolute difference between predicted and actual AV values, was calculated to assess the model’s precision.

To visually assess the performance of the CNN model in predicting AV for both the right and left hips, scatter plots were constructed. Each scatter plot depicts the relationship between the predicted AV values generated by the CNN model and the corresponding ground truth AV values obtained from CT-Scans, serving as the gold standard.

In interpreting the findings, a significance level of *P* < 0.05 was applied.

## 3 Results

Out of 300 cases, 56 (18.7%) were female. The mean age recorded was 39.45 ± 16.45 years, with a median age of 36.50 (range, 13–92).

The CNN model predicted a mean AV of 17.29 ± 5.58 degrees for the right hip, while the CT-Scan measurement yielded a mean AV of 17.19 ± 3.86 degrees (95% CI: −0.346–0.550). Notably, statistical analysis revealed no significant difference between these values (*P*-value = 0.655).

Similarly, for the left hip, the mean AV values were 16.75 ± 5.54 degrees for the CNN model and 17.15 ± 3.86 degrees for the CT-Scan measurement (95% CI: −0.839 to 0.436, *P*-value = 0.077).

In the left hip, a strong and statistically significant Pearson correlation (*r* = 0.714, *P*-value = 0.000) was observed between the AV degrees predicted by the CNN model and those measured by CT-Scan. Likewise, in the right hip, a strong and significant Pearson correlation (*r* = 0.707, *P*-value = 0.000) was found between the AV degrees predicted by the CNN model and CT-Scan measurements (Table 1).

**Table 1:** The accuracy and comparing AV among the CNN model and CT-scan.

Variables	CNN^a^ Model Predicted	CT-Scan Measurement	*P*-value*	95% CI^e^	(r^d^, *P*-value)	Female gender	Male gender	*P*-value	95% CI	SMD^c^
				(Lower, Upeer)					(Lower, Upeer)	
Right hip AV^b^ degree Mean ± SD	17.19 ± 3.86	17.29 ± 5.58	0.655	(−0.346, 0.550)	(0.707, 0.000)	20.60 ± 5.70	16.53 ± 5.28	0.000	(2.506, 5.633)	4.07
Left hip AV degree Mean ± SD**	17.15 ± 3.86	16.75 ± 5.54	0.077	(−0.839, 0.436)	(0.714, 0.000)	19.55 ± 5.20	16.1 ± 5.43	0.000	(1.870, 5.014)	3.44

^a^ CNN, Convolutional Neural Network.

^b^ AV,  Acetabular Version.

^c^ SMD, Standard Mean Difference.

^d^ *r*, Pearson Correlation Coefficient.

^e^ CI = Confidence Interval.

* *p* value < 0.05 is considered significant.

** Standard deviation.

An independent sample *T*-test showed that males (Mean = 16.1, SD = 5.43) had significantly lower left AV degrees (SMD = 3.44, 95% CI: 1.870–5.014, *P*-value = 0.000) than females (Mean = 19.55, SD = 5.20). As well as, males (Mean = 16.53, SD = 5.28) exhibited significantly lower right AV degrees (SMD = 4.07, 95% CI: 2.506–5.633, *P*-value = 0.000) compared to females (Mean = 20.60, SD = 5.70).

The MAE values for male and female participants stand at 2.92° and 3.09°, respectively. Table 2 demonstrates MAE and Pearson correlation coefficient (r) for AV prediction by gender and hip side.

**Table 2:** Mean absolute error (MAE) and Pearson correlation coefficient (r) for acetabular version prediction by gender and Hip Side.

Patients		MAE	*r*
Male (244 Patients)	Left	2.94	0.71
	Right	2.90	0.69
Female (56 Patients)	Left	3.01	0.64
	Right	3.17	0.64
Total (300 Patients)		2.95	0.71

All *p*-values were < 0.001.

The model achieved a MAE of 2.958 and 2.957 degrees for left and right hips respectively, indicating minimal deviation between predicted and actual AV values on average. Notably, over 83% of predictions exhibited errors ≤5 degrees, underscoring the model’s high degree of accuracy.

Furthermore, in the entire dataset, we found two (0.66%) cases of retroversion in the right acetabulum and one (0.33%) in the left hip. Interestingly, in all of these cases, the other acetabulum was anteverted.

**Figure 3:**
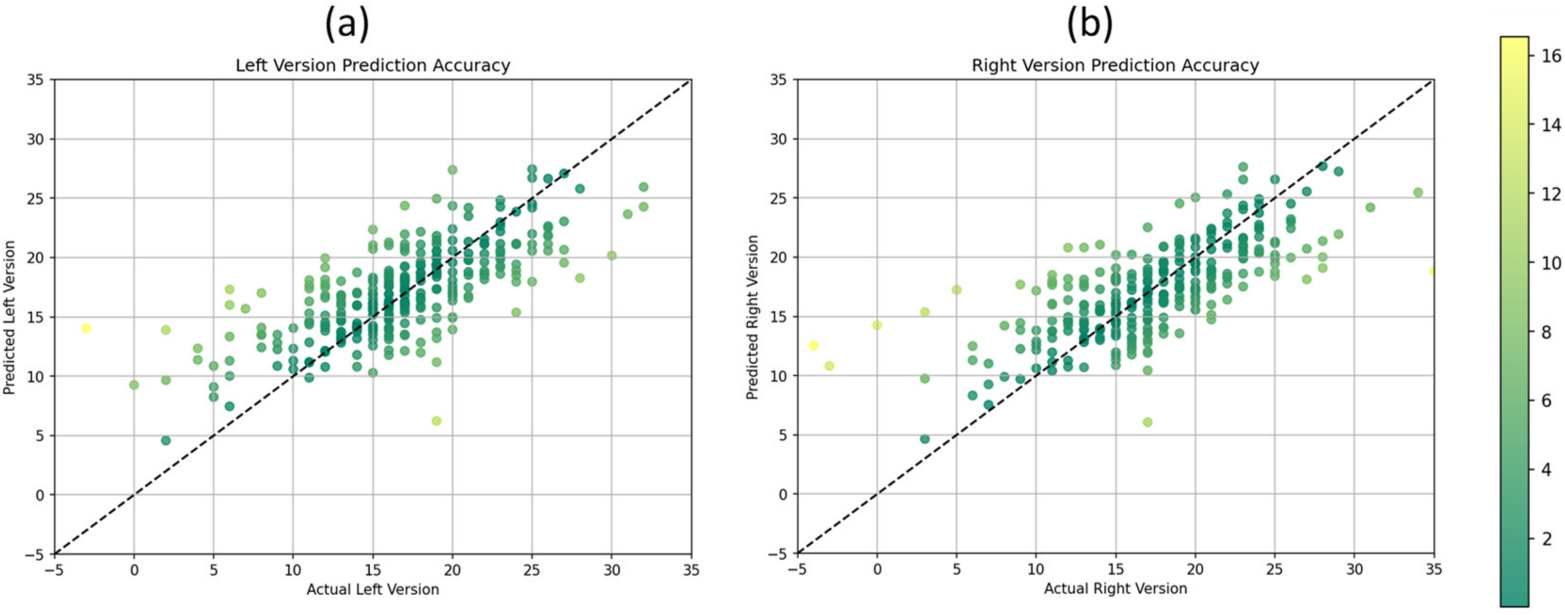
Scatter plots illustrating the relationship between predicted and actual acetabular version values for the **(a)** left version and **(b)** right version.

Figure 3 shows the scatter plots visually encapsulate the performance of the CNN model in predicting AV from plain x-ray radiographs. In areas of high frequency samples, characterized by a dense clustering of data points around the diagonal line, the model demonstrates remarkable accuracy and consistency in estimating AV values. Conversely, in outlier samples where data points deviate noticeably from the diagonal line, the model’s performance appears less optimal. Despite these occasional deviations, the majority of data points remain closely clustered, indicating overall precision and reliability in AV prediction across the dataset.

## 4 Discussion

The findings of this study underscore the efficacy of the CNN model in accurately predicting AV from AP plain radiographs of native hips. Notably, the CNN model for AV estimation achieved a MAE of ≤3° and demonstrated a strong correlation with the gold standard CT measurements. Over 83% of predictions yielded errors ≤5°, highlighting the model’s high precision. Importantly, this approach offers notable advantages over traditional CT-Scans, including reduced costs and mitigated risks associated with ionizing radiation exposure. By leveraging AP plain radiographs, the CNN model streamlines the diagnostic process while circumventing the reliance on expert interpretation and mitigating potential human errors.

Findings align with the study by Rouzrokh et al., who developed separate CNN models for AP and cross-table lateral radiographic views to measure acetabular component inclination and anteversion angles after THA (11). Their models yielded impressive results, with mean differences of only 1.35° and 1.39° from human expert measurements for inclination and anteversion, respectively. The larger training datasets (600 images per view) likely contributed to their enhanced measurement accuracy.

Despite this study’s smaller sample size, the scatter plots demonstrated a clear association between predicted and actual AV values for both hips. The distribution of data points indicates the model’s ability to capture the underlying variability in AV measurements across the dataset. Additionally, the plots illustrate the consistency of the model’s predictions across a range of AV values, as evidenced by the clustering of data points around the diagonal line, representing adequate prediction alignment. Furthermore, the scatter plots highlight the model’s performance in estimating AV with high precision. The majority of data points fall within a narrow band around the diagonal line, indicating minimal deviation between predicted and actual AV values. This tight clustering suggests that the CNN model consistently produces accurate estimations of AV, with deviations predominantly confined to a few outliers. This nuanced analysis of the scatter plots underscores both the strengths and limitations of the CNN model in accurately estimating AV from plain x-ray radiographs, providing valuable insights for its application in clinical practice.

Other investigations have explored AI techniques in orthopedic imaging, such as tools for anterior cruciate ligament (ACL) tear management (21) or measuring the center edge (CE) angle for diagnosing hip dysplasia from x-rays with high accuracy (22).

While the number of articles with using new radiographic indexes are increasing due to the x-ray benefits, they have limitations. For instance, Koyama et al. proposed a new method to quantitatively assess the acetabular version by studying *p* = the distance from the acetabular articular surface to the posterior wall and *a* = the distance from the acetabular articular surface to the anterior wall and by calculating the p/a ratio determined ante or retro version. However, this method does not measure the actual AV angle and depends on the skill of the person conducting the measurement (14). Similarly Wan and colleagues method has limited applicability and it can be only useful for AVs less than 20° (23). Whilst 3-D CT-generated models can measure AV regardless of positioning, their manual setting is nearly one hour per cases and they are time consuming (24), however our method offers a time-saving alternative, interpreting data in just two milliseconds after training.

Nitschke, A. et al. proposed a method for measuring the AV by introducing a parameter called the transverse axis distance (TAD), which showed an “excellent” correlation with a sensitivity of 0.73 and specificity of 0.82 in the assessment of ante or retro-version with CT-scan measurement (25). Similarly, Nitschke, A. et al., in another article, validated neck axis distance (NAD) as a simple, semi-quantitative radiological predictor of acetabular anteversion with an accuracy rate of 82% in comparison to CT-Scan in the prediction of retro or post-version. However, both of those articles do not provide the absolute angle of the AV as a quantitative measurement. Moreover, in both cases, operator dependency is still an issue (26).

In an earlier attempt to evaluate the AV, Jamali, et al. employed and improved a method proposed by Meunier, P. et al. (27) and used cadaveric specimens to evaluate the cranial AV, which was accurate down to 4 degrees of error, but the perplexity of this method averted physicians to utilize this method to estimate AV (26, 28). Even one of the most advanced methods in less-radiation-inducing techniques, which is EOS®, is less reliable due to its nature of standing radiography, which has been shown to alter the AV in comparison to the supine position (29).The amount of radiation is even lower than plain radiography (30), but accessibility and cost-benefit is still debated issue.

We believe that efforts to mechanize routine tasks will afford health workers more time and precision to care for patients optimally. This is particularly crucial for both inexperienced surgeons, as their dislocation rates are reported to be twice as high as their experienced counterparts (14), and experienced surgeons striving for greater precision. Studies like this one represent steps toward achieving that goal.

Interestingly, a slight gender disparity was observed, with marginally lower AV prediction accuracy for females compared to males. Despite this, the overall CNN performance remained commendable, with acceptable MAE ranges for both genders. The predominance of males (81.3%) in our dataset underscores the importance of considering gender-specific factors in model development and validation to ensure equitable outcomes across populations. Further investigations are warranted to explore potential underlying factors contributing to the observed gender differences and refine the CNN model accordingly.

## 5 Limitations

While this study demonstrates promising results with a CNN model for native hip joints, it has limitations. It focused solely on healthy joints, excluding post-operative and diseased joints affected by conditions such as osteoarthritis. The absence of retroverted acetabula in the cohort test is another limitation. Although the ratio of male and female in training and testing groups were seemingly equal it would suggest for the future researchers to include more female subjects. Additionally, the challenge of limited medical image databases, stemming from privacy issues, hampers the broader application of AI in medicine. This suggests the need for anonymized databases to improve machine learning accuracy. Other technical aspects can be addressed for example the current computational power as well as HIPPA/GDPR while using AI like so.

## 6 Conclusion

This study has successfully developed a CNN model that accurately predicts AV from AP hip plain radiographs. With a MAE ≤ 3° and achieving errors of less than five degrees in 83% of the sample population, this CNN model demonstrates remarkable precision in AV estimation. Importantly, our approach relies solely on AP hip plain radiographs, obviating the need for the conventional gold standard CT-Scan and mitigating the inherent operator dependency in angle calculation. Looking ahead, further research endeavors are warranted to refine the CNN model and ensure a comprehensive and clinically applicable assessment. Notably, our ongoing second phase involves the utilization of 5,000 unlabeled data points, employing semi-supervised learning techniques to further enhance the model’s performance and broaden its scope of application.

## Data Availability

The raw data supporting the conclusions of this article will be made available by the authors, without undue reservation.
